# Methods for Determination of Individual PEEP for Intraoperative Mechanical Ventilation Using a Decremental PEEP Trial

**DOI:** 10.3390/jcm11133707

**Published:** 2022-06-27

**Authors:** Felix Girrbach, Franziska Zeutzschel, Susann Schulz, Mirko Lange, Alessandro Beda, Antonio Giannella-Neto, Hermann Wrigge, Philipp Simon

**Affiliations:** 1 Department of Anesthesiology and Intensive Care, University of Leipzig Medical Center, 04103 Leipzig, Germany; la-franzi@gmx.de (F.Z.); susann.schulz91@gmail.com (S.S.); mirko.lange@medizin.uni-leipzig.de (M.L.); 2 Department of Surgery, Carl-von-Basedow Klinikum Saalekreis, 06217 Merseburg, Germany; 3 Department of Electronic Engineering and Postgraduate Program of Electrical Engineering, Federal University of Minas Gerais, Belo Horizonte 31270-901, Brazil; ale.beda@gmail.com; 4 Programa de Engenharia Biomédica—COPPE, Universidade Federal do Rio de Janeiro, Rio de Janeiro 21941-901, Brazil; agn@peb.ufrj.br; 5 Integrated Research and Treatment Centre (IFB) Adiposity Diseases, University of Leipzig, 04103 Leipzig, Germany; hermann.wrigge@bergmannstrost.de; 6 Department of Anaesthesiology, Intensive Care and Emergency Medicine, Pain Therapy, Bergmannstrost Hospital Halle, 06112 Halle, Germany; 7 Anesthesiology and Operative Intensive Care, Faculty of Medicine, University of Augsburg, 86161 Augsburg, Germany; arbeit.simon@gmail.com

**Keywords:** mechanical ventilation, positive end-expiratory pressure, general anesthesia, electrical impedance tomography

## Abstract

(1) Background: Individual PEEP settings (PEEP_IND_) may improve intraoperative oxygenation and optimize lung mechanics. However, there is uncertainty concerning the optimal procedure to determine PEEP_IND_. In this secondary analysis of a randomized controlled clinical trial, we compared different methods for PEEP_IND_ determination. (2) Methods: Offline analysis of decremental PEEP trials was performed and PEEP_IND_ was retrospectively determined according to five different methods (EIT-based: RVD_I_ method, Global Inhomogeneity Index [GI], distribution of tidal ventilation [EIT VT]; global dynamic and quasi-static compliance). (3) Results: In the 45 obese and non-obese patients included, PEEP_IND_ using the RVD_I_ method (PEEP_RVD_) was 16.3 ± 4.5 cm H_2_O. Determination of PEEP_IND_ using the GI and EIT VT resulted in a mean difference of −2.4 cm H_2_O (95%CI: −1.2;−3.6 cm H_2_O, *p* = 0.01) and −2.3 cm H_2_O (95% CI: −0.9;3.7 cm H_2_O, *p* = 0.01) to PEEP_RVD_, respectively. PEEP_IND_ selection according to quasi-static compliance showed the highest agreement with PEEP_RVD_ (*p* = 0.67), with deviations > 4 cm H_2_O in 3/42 patients. PEEP_RVD_ and PEEP_IND_ according to dynamic compliance also showed a high level of agreement, with deviations > 4 cm H_2_O in 5/42 patients (*p* = 0.57). (4) Conclusions: High agreement of PEEP_IND_ determined by the RVD_I_ method and compliance-based methods suggests that, for routine clinical practice, PEEP selection based on best quasi-static or dynamic compliance is favorable.

## 1. Introduction

Positive end-expiratory pressure (PEEP) during mechanical ventilation for general anesthesia is used to prevent both the formation of atelectasis as well as cyclic alveolar recruitment and de-recruitment as risk factors for ventilator-induced lung injury [1,2]. Large multi-center trials in surgical patients with an increased risk of developing postoperative pulmonary complications, however, found no difference in the primary outcome postoperative pulmonary complications in non-obese and obese patients, if constant PEEP levels of ≤5 cm H_2_O or 12 cm H_2_O were compared [3]. Individually titrated PEEP levels aiming at avoiding atelectasis and cyclic recruitment have been shown to vary significantly due to patient factors such as respiratory system mechanics, obesity, distribution of fat tissue, and procedural factors. The latter include, for example, laparoscopic abdominal surgery and patient positioning, which may vary between reverse Trendelenburg position, e.g., during bariatric surgery, or steep Trendelenburg position, e.g., during prostatic surgery [4,5].

We have recently shown that an individualized PEEP (PEEP_IND_) titration using electric impedance tomography (EIT) in obese patients resulted in a PEEP range of 10–26 cm H_2_O with a median of 18 cm H_2_O when selecting PEEP according to the lowest temporal inhomogeneity determined by the regional ventilatory delay (RVD_I_ method) [6]. The comparison with a subgroup of patients of the large PROBESE trial comparing standard higher PEEP of 12 cm H_2_O vs. PEEP of 4 cm H_2_O suggested that lung recruitment was incomplete in both arms of the PROBESE trial [7]. Likewise, even in non-obese patients, the PEEP_IND_ determined by EIT was found as high as median of 14 cm H_2_O (median, range of 8–20 cm H_2_O) [4]. While these and other results [8,9] may favor the concept of an individualized ventilation strategy in interventions with high risk of pulmonary complications, there is uncertainty concerning the optimal procedure to determine PEEP_IND_. Regardless of the method chosen, a decremental PEEP trial preceded by a recruitment maneuver to reopen the lung is usually performed for the determination of PEEP_IND_ [8,9,10].

Due to its simplicity and the lack of need for additional equipment, identification of the optimal individual PEEP level based on dynamic or static compliance is widely used in the OR and ICU [10,11,12,13,14]. However, it remains unclear how high the agreement between the different methods used to identify PEEP_IND_ is.

In this secondary analysis, we therefore compared different methods for the determination of PEEP_IND,_ including both parameters assessing global parameters of the respiratory system and EIT-based parameters. Accordingly, we included patients from the intervention group of a two-part, prospective, randomized controlled trial in obese and non-obese patients [4,6].

## 2. Materials and Methods

We retrospectively analyzed the data of the intervention groups (obese and non-obese patients) of a randomized controlled study dealing with individual PEEP titration using EIT in obese and non-obese patients [4,6]. The study (German clinical trials register No. DRKS00004199, www.who.int/ictrp/network/drks2/en/ accessed in 25 June 2022) was conducted at the University Hospital of Leipzig Medical Center. Approval for the trial was granted by the Leipzig University Ethics Committee (No. 196-11-ff-8042011) and all patients gave written informed consent prior to inclusion. Detailed description of the methods are published [4,6].

### 2.1. Patients

Between November 2012 and August 2017, non-obese patients with a body mass index (BMI) between ≥18.5 and <30 kg/m^2^, and obese patients with a BMI ≥ 35 kg/m^2^, age ≥ 18 years, and with a medium or high risk of postoperative pulmonary complications (“Assess Respiratory RIsk in Surgical patients in CATalonia, ARISCAT score” ≥ 26 [15,16]) scheduled for elective laparoscopic surgery were previously included. All patients included in the present secondary analysis were assigned to the intervention group of the original two-part study and received intraoperative mechanical ventilation with an individualized PEEP after an initial recruitment maneuver.

### 2.2. PEEP Titration and Determination of PEEP_RVD_

After induction of anesthesia and intubation, all patients were ventilated at a PEEP of 5 cm H_2_O for ten minutes before baseline measurements were performed. All patients were ventilated with a standard intensive care unit respirator (Evita-XL, Dräger Medical AG, Lübeck, Germany).

Before the start of surgery and before insufflation of pneumoperitoneum, all patients received an initial recruitment maneuver (RM). The RM was performed in pressure-controlled mode, by gradually increasing PEEP and peak inspiratory pressure (PIP) until 30 cm H_2_O and 50 cm H_2_O, respectively, for the obese group (20 cm H_2_O and 40 cm H_2_O for the non-obese group). These settings were then maintained for ten respiratory cycles at a respiratory rate of 6/min with an inspiratory to expiratory ratio of 1:2. The RM was followed by a decremental PEEP trial for determination of PEEP_IND_ [17]. The decremental PEEP trial was performed in volume-controlled mode with a respiratory rate of 12/min. Inspiratory to expiratory ratio of 1:2 and a tidal volume of 8 mL/kg predicted body weight. Inspiratory time was set to achieve an inspiratory pause > 0.2 s to reduce the influence of resistance on compliance calculation. The decremental PEEP trial started with a PEEP of 26 cm H_2_O in obese patients or 20 cm H_2_O in non-obese patients, and was decreased stepwise by 2 cm H_2_O until a PEEP level of 4 cm H_2_O was reached, with an interval of 3 min at each PEEP step [4,6]. PEEP titration was performed using a 20° head-elevated ramped position in obese patients and a 30° Trendelenburg position in non-obese patients. At the end of each PEEP step, a single low-flow inflation maneuver was performed (tidal volume 12 mL/kg PBW, inspiratory flow 4 L/min). The Regional Ventilatory Delay Index (RVD_I_) during each low-flow maneuver was subsequently calculated offline using customized software.

**Figure 1:**
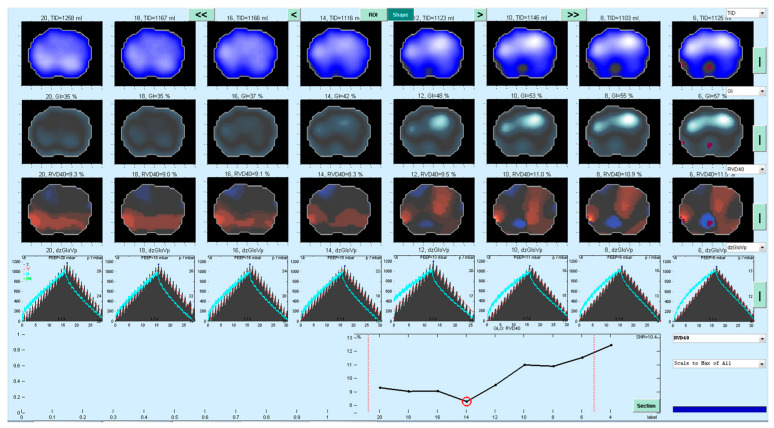
Example of a decremental PEEP trial and identification of PEEP_IND_ using the RVD_I_ method, illustrating the PEEP steps 20–6 cm H_2_O. Distribution of tidal ventilation significantly shifts to nondependent (ventral) lung areas with decreasing PEEP levels (top row). RVD_I_ reaches its minimum at a PEEP level of 14 cm H_2_O (third row of images from top, line graph at the bottom). Likewise, Global Inhomogeneity Index (second row of images from top significantly increases below PEEP levels of 14 cm H_2_O. Maximum dynamic compliance was reached at a PEEP level of 16 cm H_2_O and was 152 mL/cm H_2_O.

As described in a previous publication [18], the Regional Ventilatory Delay (*RVD*) [19] for each pixel in the EIT image was determined during the low-flow maneuver. In brief, the *RVD* describes the delay (given in [%] of inflation time of the LFM) until each single pixel’s regional impedance change exceeds 40% of its respective impedance maximum in the impedance–time curve (Formula (1)). The RVD_I_ is then defined as the SD for all pixels’ regional ventilatory delay [18]. An example is given in Figure 1. The PEEP corresponding to the lowest RVD_I_ was identified as the individual PEEP as determined by the *RVD* method (PEEP*_RVD_*) [18].

(1) $$RVD=\frac{\Delta ti^{40\%}}{t_{max}-t_{min}}\times 100\%$$

During the decremental PEEP trial, ventilation and respiratory parameters at every PEEP level were measured after the equilibration phase and before the low-flow maneuver, transferred to a PC, and stored for offline analysis. Likewise, quasi-static compliance in the inspiration and expiratory phase of the low-flow maneuver was recorded. Ventilation distribution images were obtained with a commercially available EIT system (PulmoVistaTM, Dräger Medical AG, Lübeck, Germany). In addition, regional ventilation distribution and homogeneity using the global inhomogeneity index (GI) [20], and the percentage of tidal volume distributed to the non-dependent lung areas (relative amount of ventilated pixels in dorsal half compared with all ventilated pixels in the EIT-image) were quantified at every PEEP step.

### 2.3. Comparative Analysis of Different Methods to Determine PEEP_IND_

#### 2.3.1. EIT Derived Parameters (PEEP_GI_, PEEP _EIT VT_)

The Global Inhomogeneity Index was developed to quantify the tidal volume distribution within the lung [20]. For calculation of GI, the median value of impedance differences between end-inspiration and end-expiration is determined. Thereafter, the sum of the absolute difference between median value and every pixel value is calculated and divided by the sum of impedance values within the lung (Formula (2)). The *GI* was automatically calculated at each PEEP step by our customized software and PEEP*_GI_* was defined as the PEEP level with the lowest *GI*.

(2) $$GI=\frac{\sum_{x,y\;\in \;lung}^{}\left|DI_{xy}-Median(DI_{lung})\right|}{\sum_{x,y\;\in \;lung}^{}DI_{xy}}$$

*DI*: value of the differential impedance in the tidal images, *DI_xy_*: the pixel in the identified lung area, *DI_lung_*: all pixels representing the lung area, and ∈: element of the ventilated part of the lung [20].

PEEP _EIT VT_ was defined as the PEEP level where ventilation distribution between the two ventral and the two dorsal regions of interest in the EIT-image was balanced.

#### 2.3.2. Global Parameters of the Respiratory System (PEEP _Cdyn_, PEEP _CQstat_)

Dynamic compliance (C_dyn_) was determined during the last five breaths of each PEEP level of the decremental PEEP trial. Additionally, quasi-static compliance (C_Qstat_) was determined during the initial part of the inspiratory phase of the low-flow maneuver (from start until a volume equal to VT was reached) at each PEEP step. Both C_dyn_ and C_Qstat_ were calculated offline from airflow (F), airways pressure (P), and respiratory volume (V) waveforms using the least squares method to estimate compliance (C) and resistance (R), according to the equation of motion (P = R*F + V/C + PEEP). PEEP C_dyn_ was defined as the PEEP level showing the highest dynamic compliance and PEEP C_Qstat_ as the PEEP level showing the highest quasi-static compliance. Instead of calculating compliance of the respiratory system offline, the compliance values estimated by the ventilator are often used in clinical routines for PEEP titration. We therefore also compared the compliance values determined offline with the values read from the respirator.

While the RVD is determined using a low-flow maneuver and is therefore linked to quasi-static compliance, the GI is determined during tidal ventilation and is therefore more linked to dynamic compliance. For the sake of clarity, we therefore limited ourselves to comparing the PEEP_RVD_ with the PEEP _CQstat_ and PEEP_GI_ with PEEP _Cdyn_. Furthermore, the two most popular EIT-based methods (PEEP_GI_ and PEEP_RVD_) were each compared separately with the PEEP_EIT VT_.

### 2.4. Statistical Analysis

The data are presented as mean with standard deviation or median with range, except for sex, where the number is given. The comparison of the PEEP_IND_ obtained with different methods compared to the PEEP_IND_ used according to RVD_I_ was performed according to the method by Bland–Altman [21]. Clinically significant diverging results of PEEP selection among the measures were defined as differences > 2 PEEP steps (i.e., >4 cm H_2_O). The statistical significance between the methods was determined with the Student’s *t*-test if normally distributed. Otherwise, the Wilcoxon signed-rank test was used. Regression analyses were performed by fitting a linear model. Normal distribution was tested by the Shapiro–Wilk test and by plotting QQ plots. All statistical analyses were performed using R Version 3.6.1 (R Foundation for Statistical Computing, Vienna, www.r-project.org accessed on 25 June 2022, and RStudio, Version 1.2.1335). All tests were two-tailed and *p* < 0.05 was considered to be statistically significant. 

## 3. Results

**Figure 2:**
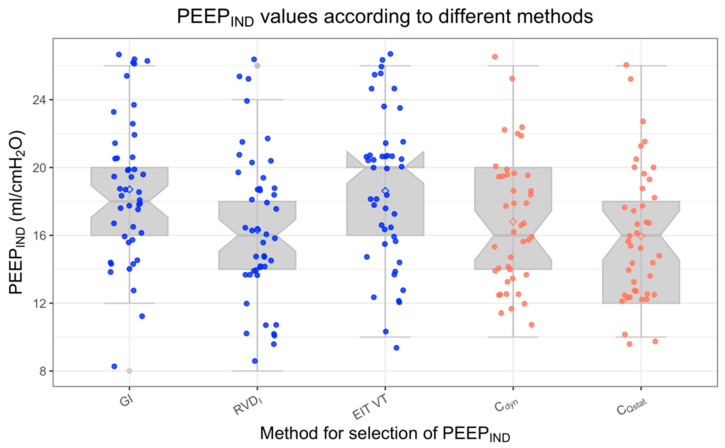
PEEP_IND_ values according to different methods for identification of PEEP_IND_. Median PEEP values did not differ between the RVD_I_ method and quasi-static compliance measured during the inspiratory limb of the LFM. However, median PEEP_IND_ values according to the minimal Global Inhomogeneity Index (GI) were around 2 cm H_2_O higher than according to the other methods.

A total of 45 patients were included in this secondary analysis (20 patients with a BMI ≥18.5 and <30 kg/m^2^, and 25 patients with a BMI ≥ 35 kg/m^2^). Based on the surgical interventions, intraoperative positioning was 30° Trendelenburg in non-obese patients and in reverse Trendelenburg position in obese patients. Baseline characteristics of the study group are presented in Table 1. Mean PEEP_IND_ according to the RVD_I_ method did not statistically differ between obese and non-obese patients (*p* = 0.09, Table 1). Data of the decremental PEEP trial concerning RVD_I_ were available for all patients assigned to the intervention group in the primary trials (*n* = 45), whereas data concerning the quasi-static compliance during the inspiratory or expiratory limb of the low flow maneuver and data for analysis of dynamic compliance were unavailable in *n* = 3 patients. The mean PEEP_IND_ values according to the different methods are summarized in Figure 2.

### 3.1. Determination of PEEP_IND_ Using EIT-Based Parameters

As presented in Table 2 and Figure 2, determination of PEEP_IND_ using the Global Inhomogeneity Index and the ventral-to-dorsal distribution of tidal ventilation (EIT VT) led to significantly higher PEEP values compared to the RVD_I_ method, with a mean difference of −2.4 cm H_2_O (95% CI: −1.2; −3.6 cm H_2_O, *p* = 0.01) for the GI method and −2.3 cm H_2_O (95% CI: −0.9;3.7 cm H_2_O, *p* = 0.01) for the EIT VT method. Potentially relevant diverging PEEP_IND_ values compared to the RVD_I_ method with an absolute deviation of >4 cm H_2_O were evident in 10 patients (22%, GI method) vs. 13 patients (29%, EIT VT method). 

### 3.2. Compliance-Based Parameters

Overall, the mean resulting PEEP_IND_ values derived from the different methods searching for the highest dynamic compliance of the respiratory system (C_dyn_) or the highest quasi-static compliance estimated during a standardized low-flow breath of 12 mL/kg PBW (C_Qstat_) did not significantly differ from the mean PEEP_IND_ values determined with the RVD_I_ method (Table 2 and Figure 2).

PEEP_IND_ determination according to the maximum dynamic compliance during normal tidal ventilation at the decremental PEEP trial resulted in PEEP values with a divergence of >4 cm H_2_O in 5/42 patients (12%, two obese patients and three non-obese patients).

**Figure 3:**
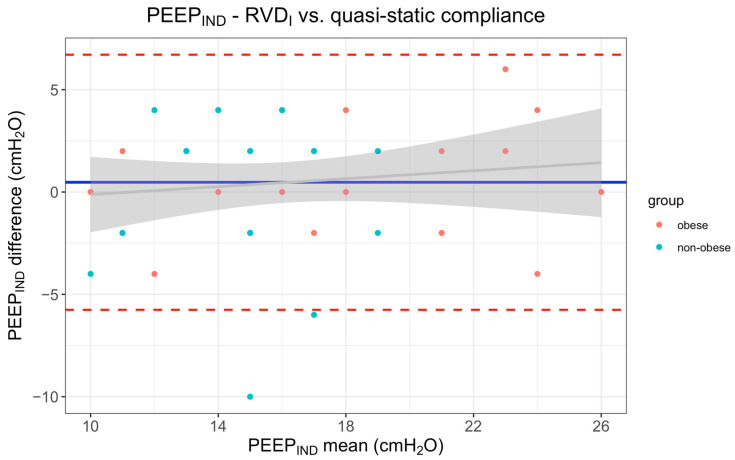
Bland–Altman plot between PEEP_IND_ using regional ventilation delay index (RVD_I_) and PEEP_IND_ determination using quasi-static compliance as calculated by the least squares method (C_Qstat_). Bias was 0.5 ± 3.18 cm H_2_O and did not differ between obese and non-obese patients (0.7 ± 2.7 cm H_2_O vs. 0.2 ± 3.7 cm H_2_O, *p* = 0.99). Additional Bland-Altman plots comparing RVD_I_, GI and compliance-based methods can be found in the Supplemental Material.

Potentially relevant deviations in PEEP_IND_ > 4 cm H_2_O were found in three patients when comparing the RVD_I_ method and the C_Qstat_ method (Figure 3). One of these patients had a BMI of 65 kg/m^2^, which was the highest of the entire study population. In the other two patients, re-examination of the PEEP titration curve revealed a partially erroneous recording. 

**Figure 4:**
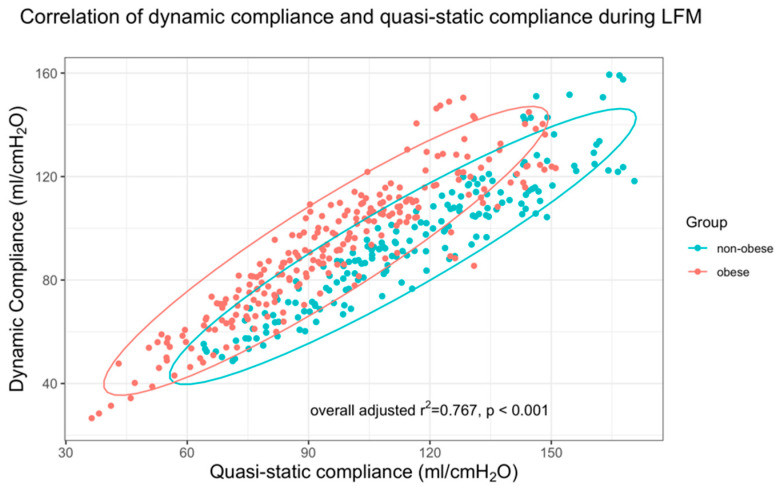
Correlation between dynamic compliance and quasi-static compliance of the respiratory system during the inspiratory limb of the low-flow maneuver (LFM)-based on the data calculated using the least squares method. Dynamic compliance and quasi-static compliance during the LFM highly correlated in both obese and non-obese patients.

Calculated dynamic compliance during tidal ventilation at each PEEP step using the least squares method showed a strong correlation to the inspiratory compliance during the standardized low-flow inspiratory breath (r^2^ = 0.767, *p* < 0.001, Figure 4). Bland–Altman statistics showed a bias of 11.0 ± 13.4 mL/cm H_2_O with a precision (or CI) of 1.28 mL/cm H_2_O. Concerning the resulting levels of PEEP_IND_, bias was −0.9 ± 2.4 cm H_2_O with a precision of 0.74 cm H_2_O.

**Figure 5:**
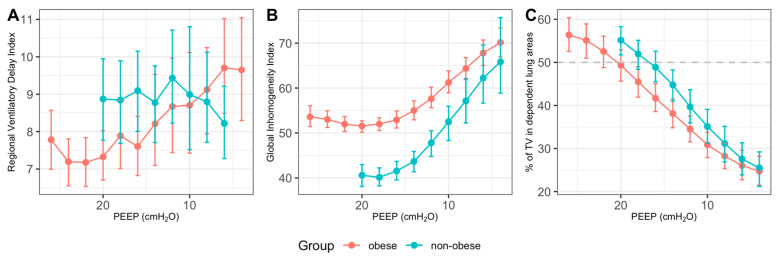
Course of mean Regional Ventilatory Delay Index (**A**), mean Global Inhomogeneity Index and mean distribution of tidal ventilation to dependent (dorsal) lung areas in obese (red) and non-obese patients (green) during the decremental PEEP trial. While RVD shows considerable interindividual variation at each PEEP step (**A**), GI (**B**) is minimal at a PEEP of 18 cm H_2_O in normal weighted patients and at 20 cm H_2_O in obese patients. Likewise, best PEEP values according to the TV distribution method show best PEEP values at 20 cm H_2_O for obese patients and 16 cm H_2_O for non-obese patients (**C**).

Figure 5 depicts the course of mean Regional Ventilatory Delay Index (A), mean Global Inhomogeneity Index and mean distribution of tidal ventilation to dependent (dorsal) lung areas in obese (red) and normal weighted patients (green) during the decremental PEEP trial, which show a homogeneous course of both the GI and VT values in both groups. Mean PEEP_IND_ values using the GI were 20.1 ± 4.6 cm H_2_O (obese patients) vs. 17.0 ± 3.3 cm H_2_O (normal weighted patients), while mean PEEP_IND_ values of the VT distribution method were 20.4 ± 4.4 cm H_2_O (obese patients) vs. 16.4 ± 3.6 cm H_2_O (normal weighted patients).

## 4. Discussion

The main result of the present study comparing global parameters of the respiratory system and parameters derived by EIT is that PEEP_IND_ values derived by the two EIT-based methods (Global Inhomogeneity Index and Distribution of Tidal Ventilation) tended to be slightly higher than PEEP_IND_ values derived by compliance-based methods and the RVD_I_ method. However, mean PEEP values ranged from 16.0 cm H_2_O to 18.7 cm H_2_O, all determined with different methods, and were thus significantly higher than the PEEP values usually applied during the surgical interventions examined, both in obese and normal weighted patients and in different body positions with pneumoperitoneum [22,23].

Pneumoperitoneum during laparoscopic surgery leads to a cephalad shift of the diaphragm and decreases transpulmonary pressure, and therefore leads to airway closure and formation of atelectasis in dependent lung areas, especially when combined with a Trendelenburg position [24,25,26,27,28]. Intraoperative mechanical ventilation with a lung-protective strategy consisting of adequate PEEP—possibly adapted to the pressure of the pneumoperitoneum—and repeated recruitment maneuvers can counterbalance these effects [26,27,29,30]. This leads to lower elastance of the respiratory system and consecutively improves oxygenation during surgery [28,30,31]. Many authors have advocated the individualization of PEEP in the operating room [32,33], and there is recent evidence that individualized PEEP is superior to a standardized “one PEEP fits all” strategy, even if higher PEEP levels are routinely used [7]. The optimal method for the choice of best PEEP during mechanical ventilation in the operating room is, however, less clear [34].

Today, several methods to determine individual PEEP levels based on a decremental PEEP trial have been proposed. Advanced methods aim to optimize lung mechanics, gas exchange or the regional distribution and homogeneity of pulmonary ventilation, which can be directly measured by Electrical Impedance Tomography (EIT). EIT-based regional-ventilation-delay inhomogeneity has shown to be well correlated with the amount of tidal recruitment [18] and mechanical ventilation, with a PEEP_IND_ identified using the Regional Ventilatory Delay Index (RVD_I_) resulting in better intraoperative oxygenation in patients undergoing high risk surgery for postoperative pulmonary complications [4,6].

The various methods investigated in the present study can be divided into those aimed at optimizing the mechanics of the entire respiratory system and those seeking to homogenize pulmonary ventilation to the greatest extent possible. Our reference method, and at the same time the method used in the original two-part study [4,6], is to minimize the RVD_I_ measured by EIT during a standardized, low flow breath. This enables the quantification of the delayed aeration of (mainly dependent) lung areas if PEEP is set below the closing pressure of those lung units [19]. In this situation, the opening pressure of lung units that are collapsed in the end-expiratory state is exceeded by inspiratory pressure during the low flow breath, which then presents as a regional delayed change in impedance in electrical impedance tomography. Minimizing this temporal inhomogeneity of regional ventilation by setting PEEP according to the lowest RVDI [3,4,6,19] should thus result in the lowest PEEP that minimizes tidal alveolar collapse, consequently helping to prevent atelectrauma in dependent lung areas and volutrauma in non-dependent lung areas. In contrast, methods based on finding maximum compliance—either during tidal ventilation or during a standardized LFM of the decremental PEEP trial—only allow global assessment of the respiratory system and do not directly pay attention to regional pulmonal heterogeneity. Atelectasis and overdistension may occur simultaneously and may only be detected when regional information is available. To prevent overdistension and consecutive volutrauma in non-dependent lung areas, additional regional information is thus necessary. Although the RVD_I_ method is validated by computed tomography [19], it is has been rarely used so far for determination of PEEP_IND_. Blankman et al. [35] also compared different methods for determination of PEEP_IND_, but were not successful in defining an optimal PEEP level during a four-step decremental PEEP trial using the GI and the RVD_I_ method in a cohort of 12 post-cardiac surgery patients. However, the highest PEEP applied in the study from Blankman et al. was only 15 cm H_2_O, which was 1.3 cm H_2_O lower than the mean PEEP_IND_ determined by the RVD_I_ method in our patient cohort, so the optimal PEEP level might have been missed. Moreover, the RVD_I_ was calculated during tidal ventilation instead of performing a low-flow maneuver, which may have contributed to these negative results. Muders et al. recently found that the tidal volume for low-flow breathing can be reduced down to 6 mL/kg PBW, avoiding possible overdistension at higher PEEP levels during the decremental PEEP trial. However, EIT-based selection of PEEP_IND_ was not possible on the basis of RVD_I_ values derived from regular tidal ventilation [36], which could additionally explain the differing results of the Blankman study.

Due to its ability to assess regional ventilation changes during a decremental PEEP trial, EIT has become increasingly interesting for determination of an individualized PEEP. Alternative methods used in addition to the RVD_I_ method include the Global Inhomogeneity Index (GI) [11,20,37] and homogenization of ventilation distribution between dependent and non-dependent lung areas [37]. In our patient cohort, both methods led to slightly but significantly higher PEEP_IND_ values compared to the RVD_I_ method, and we observed differences in PEEP_IND_ up to 14 cm H_2_O. Zhao et al. also observed large differences concerning PEEP_IND_ in 10% of patients with ARDS undergoing a decremental PEEP trial when using different methods [11]. However, they used a less restrictive definition of significantly differing PEEP values and tolerated deviations up to 8 cm H_2_O. Heterogeneous resistance of the respiratory tract and non-recruitable lung areas were discussed as causes for the large deviations. In line with our results, the PEEP_IND_ values based on the GI were approximately 2 cm H_2_O higher than the PEEP_IND_ values based on global parameters (quasi-static or dynamic compliance). The GI might therefore slightly overestimate the optimal individual PEEP level, which could be due to the GI not taking into account the presence of atelectasis and overdistension [35]. Likewise, assessing the ventral-to-dorsal distribution of tidal ventilation is similarly insensitive for overdistension, and PEEP selection based on ventral-to-dorsal distribution of tidal ventilation resulted in similar mean PEEP_IND_ values compared to the GI method in our patient cohort (Figure 2).

The main advantage of best PEEP selection based on respiratory system compliance is that it does not require additional equipment. In our patient cohort, we found a sufficient agreement between PEEP_IND_ based on best dynamic compliance and PEEP_IND_ based on the RVD_I_ method in 88% of the patients. When PEEP_IND_ was selected based on the best quasi-static compliance, there was a sufficient agreement with PEEP_RVD_ in 93% of the patients in our study cohort. PEEP_IND_ selection based on quasi-static compliance is therefore the preferred method if regional information (EIT) is not available. In addition, in patients with healthy lungs, a compliance-based estimation of PEEP_IND_ seems to provide sufficiently accurate results according to our data and can be more easily performed than EIT in the routine operative setting. However, while determination of PEEP_IND_ based on compliance may be reasonably accurate in patients with healthy lungs, the data published by Zhao and colleagues also indicate that EIT provides valuable regional information in patients with diseased lungs and significant inhomogeneity of ventilation distribution [11], or, more generally, information for answering scientific questions.

Furthermore, Zhao et al. found in their feasibility study that best PEEP selection based on the GI was not superior to the compliance-based method in patients with healthy lungs [37]. This may partly be explained by a high signal-to-noise ratio of EIT in healthy patients. Overall small absolute values of RVD_I_ and small changes in RVD_I_ with a high signal-to-noise ratio were also discussed as a reason for a disproportionally high number of RM in almost completely recruited lungs in a model of experimental ARDS, resulting in mean SD_RVD_ values of ~5 [38]. Therefore, this may also have affected our results in patients with healthy lungs, where at higher PEEP steps an almost completely recruited lung may have affected the discriminatory power of the EIT-based parameters, especially the RVD_I_ (Figure 5). Nevertheless, this emphasizes the advantage of an RVD-based analysis, which, in addition to the interpretation of a pure numerical value, allows the visual analysis and thus the detection of hyperinflation of individual lung segments (Figure 1). However, large deviations of PEEP_IND_ values derived by the RVD_I_ method and compliance-based methods should trigger a re-examination of the PEEP titration and the EIT measurements as they may indicate a faulty measurement. In our study, we detected large deviations of PEEP_IND_ in three patients. One of these patients had a BMI of 65 kg/m^2^, which was the highest BMI in our population. Hence, a reason for the large deviation in this patient could be that the EIT reconstruction algorithm reaches its limits in this case. Additionally, it could be useful to check the PEEP_IND_ values determined for plausibility by means of a second method, e.g., the quasi-static or dynamic compliance.

Dynamic compliance highly correlated with quasi-static compliance in our patient cohort (Figure 4). A low-flow maneuver therefore seems dispensable if no RVD_I_ is determined. However, to eliminate the effect of the endotracheal tube and airways on the calculation of dynamic compliance, care should be taken that there is an inspiratory pause during mechanical ventilation and to use inspiratory plateau pressure instead of peak inspiratory pressure for compliance estimation.

The present study was a retrospective analysis of experimental data comparing the results of different methods for determination of PEEP_IND_. Although PEEP_IND_ values were in many cases within the previously defined range of maximum 4 cm H_2_O deviation, some patients showed larger deviations of PEEP_IND_ values, depending on the chosen method. Because we did not prospectively compare the different methods of PEEP_IND_ determination and patients in the study were always ventilated with the PEEP_IND_ based on the RVD_I_ method in the primary study, we cannot make a definitive statement as to whether the results of the primary study would have differed with respect to the achieved intraoperative PaO_2_/FiO_2_ values and pulmonary mechanics if PEEP_IND_ values based on other methods had been chosen. In particular, regarding the secondary endpoint “driving pressure”, the difference could be negligible because it did not change significantly over several PEEP steps in most patients.

As discussed in the original publication [4], practical issues that may still hinder the application of an EIT-based PEEP strategy in the operating room are the limited availability of the method, the need for a time-consuming PEEP trial, and that some anesthesia machines may not allow setting PEEP values higher than 20 cm H_2_O. Further research should therefore also focus on EIT-based methods of PEEP_IND_ findings that do not require a decremental PEEP trial.

## 5. Conclusions

We found that, independently of the method used for determination of PEEP_IND_, individualized PEEP values are significantly higher than the PEEP values usually applied during laparoscopic surgery, both in obese and non-obese patients. In most patients, the different methods show sufficient agreement of the resulting PEEP_IND_ values, and large deviations between different methods may indicate confounding factors. For clinical routine practice, PEEP selection based on best quasi-static or dynamic compliance during a decremental PEEP trial is favorable and showed the highest agreement with the PEEP_IND_ determined by the RVD_I_ method.

## Figures and Tables

**Table 1:** Patient characteristics and PEEP_IND_ according to the RVD_I_ method. Entries are mean (standard deviation) or numbers. * *p* < 0.05.

	All Patients	Non-Obese	Obese	*p*-Value
Number	45	20	25	
Age (years)	52.8 (12.7)	62.6 (7.5)	44.9 (10.3)	<0.001 *
Sex (male/female)	28/17	20/0	8/17	<0.001 *
Height (cm)	177 (11)	182 (9)	173 (11)	0.004 *
Weight (kg)	118.0 (36.4)	84.4 (12.2)	145.0 (24.6)	<0.001 *
BMI (kg m^2^)	38.1 (12.7)	25.4 (2.3)	48.2 (7.0)	<0.001 *
PEEP_IND_ RVD_I_ (cm H_2_O)	16.3 (4.5)	14.9 (3.1)	17.4 (5.2)	0.047 *

**Table 2:** Comparison of different methods for PEEP_IND_ determination during a decremental PEEP trial compared to the regional ventilation delay index method (RVDI) using Electrical Impedance Tomography (EIT); data are shown as mean with 95% CI or number (percent). * *p* < 0.05.

Method	n	Mean PEEP_IND_ (95% CI)	n with Difference to PEEP RVD_I_ max. 4 cm H_2_O	Mean Difference to PEEP RVD_I_ (95% CI)	*p*-Value
RVD_I_	45	16.3 (14.9–17.6)	–	–	–
EIT GIT	45	18.7 (17.4–20.0)	35 (78%)	−2.4 (−1.2; −3.6)	0.010 *
EIT VT	45	18.6 (17.3–19.9)	32 (71%)	−2.3 (−0.9; −3.7)	0.014 *
C_dyn_	42	16.8 (15.6–18.0)	37 (88%)	−0.4 (0.7; −1.5)	0.57
C_Qstat_	42	16.0 (14.8–17.1)	39 (93%)	0.5 (1.5; −0.5)	0.67

## Data Availability

The data presented in this study are available from the corresponding author (F.G.), upon reasonable request.

## Supplementary Materials

The following supporting information can be downloaded at: https://www.mdpi.com/article/10.3390/jcm11133707/s1, Figure S1: Bland–Altman plots for comparison of different methods for PEEP_IND_ determination during a decremental PEEP trial.
