# Supplementary material for: Methods for Determination of Individual PEEP for Intraoperative Mechanical Ventilation Using a Decremental PEEP Trial
Source: J Clin Med. 2022 Jun 27;11(13):3707. doi: 10.3390/jcm11133707 (PMC9267263; doi:10.3390/jcm11133707)
Supplement: Supplementary file 1 [file jcm-11-03707-s001.zip › jcm-1681044-supplementary.pdf]

Journal of Clinical Medicine

MDPI

Supplementary Materials

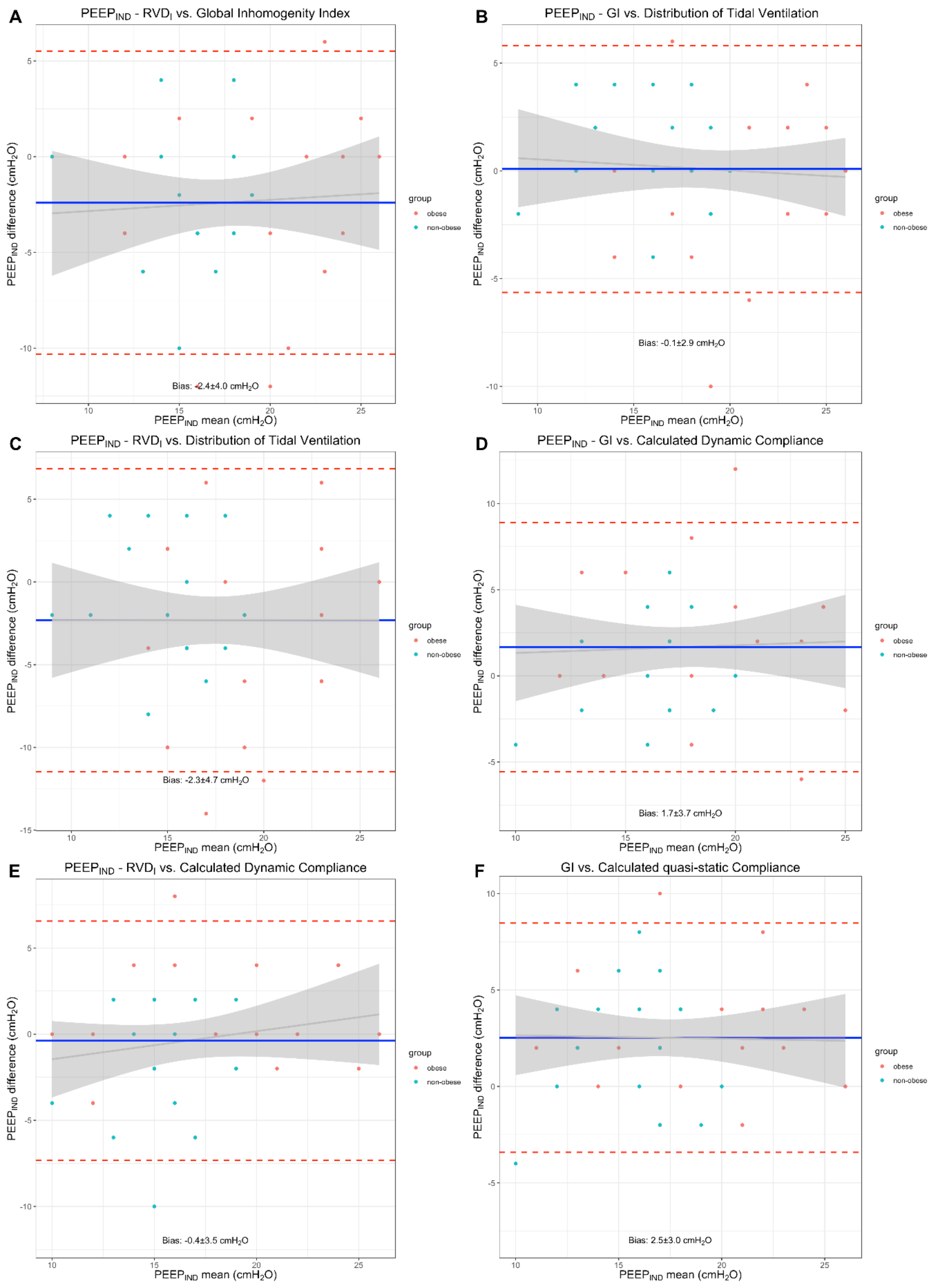

Figure S1. Bland-Altman-plots for comparison of different methods for $PEEP_{IND}$ determination during a decremental PEEP trial.
